# Genetic regulation of *THBS1* methylation in diabetic retinopathy

**DOI:** 10.3389/fendo.2022.991803

**Published:** 2022-11-14

**Authors:** Yaqi Li, Chunmei Gong, Yuanfei Xu, Xiongshun Liang, Xiaoping Chen, Wenxu Hong, Junxia Yan

**Affiliations:** ^1^ Department of Epidemiology and Health Statistics, XiangYa School of Public Health, Central South University, Changsha, Hunan, China; ^2^ Animal Laboratory, Shenzhen Center for Chronic Disease Control, Shenzhen, China; ^3^ Central Laboratory, Shenzhen Center for Chronic Disease Control, Shenzhen, China; ^4^ Institute of Clinical Pharmacology, Central South University, Changsha, China; ^5^ Hunan Provincial Key Laboratory of Clinical Epidemiology, XiangYa School of Public Health, Central South University, Changsha, Hunan, China

**Keywords:** meQTL, DNA methylation, *THBS1*, diabetic retinopathy, genetic regulation

## Abstract

**Background:**

Diabetic retinopathy (DR) is a common and serious microvascular complication of diabetes mellitus (DM), but its pathological mechanism, especially the formation mechanism of new blood vessels remains unclear. Thrombospondin-1 (*THBS1*) is a potent endogenous inhibitor of angiogenesis and it was found over expressed in DR in our previous study. Our study aimed to determine whether overexpression of *THBS1* is associated with its promoter methylation level, and whether methylation of *THBS1* is regulated by genetic variants in DR.

**Methods:**

Patients diagnosed with DR and DM patients without retinal problems were included in the case-control study. DNA methylation detection of *THBS1* by bisulfite sequencing and genotyping of specific SNPs by MassARRAY analysis were performed in the patients recruited from 2019-2020. Real time quantitative PCR was performed to obtain mRNA expression of *THBS1* in the patients recruited from August to October 2022. The differentially methylated CpG loci of *THBS1* were identified by logistic regression, and associations between 13 SNPs and methylation levels of CpG loci were tested by methylation quantitative trait loci (meQTLs) analysis. Mediation analysis was applied to determine whether CpG loci were intermediate factors between meQTLs and DR.

**Results:**

150 patients diagnosed with DR and 150 DM patients without retinal complications were enrolled in the first recruitment, seven DR patients and seven DM patients were enrolled in the second recruitment. The patients with DR showed promoter hypomethylation of *THBS1* (P value = 0.002), and six out of thirty-nine CpG sites within two CpG islands (CGIs) showed hypomethylation(P value < 0.05). *THBS1* mRNA expression in peripheral blood was significantly higher in DR patients than in DM patients. Five out of thirteen cis-meQTLs were identified to be associated with CpG sites: rs13329154, rs34973764 and rs5812091 were associated with cis-meQTLs of CpG-4 (P value=0.0145, 0.0095, 0.0158), rs11070177 and rs1847663 were associated with cis-meQTLs of CpG-2 and CpG-3 respectively (P value=0.0201, 0.0275). CpG-4 methylation significantly mediated the effect of the polymorphism rs34973764 on DR (B=0.0535, Boot 95%CI: 0.004~0.1336).

**Conclusion:**

*THBS1* overexpression is related to *THBS1* hypomethylation in patients with DR. DNA methylation may be genetically controlled in DR.

## Introduction

Diabetic retinopathy (DR) is a common and serious microvascular complication of diabetes mellitus (DM), and has long been regarded as one of the major public health problems in the world (1, 2). An up-to-date evaluation report showed that one in five patients with DM worldwide had DR, and the number of adults with DR worldwide was estimated to rise from 103.12 million in 2020 to 160.5 million in 2045 (3). Clinically, DR not only leads to visual impairment and blindness, but also signifies an enhanced risk of cardiovascular disease, atherosclerosis and diabetic peripheral neuropathy (4–7). Glycemic control is routinely recommended to prevent the complications of DM. However, multiple basic studies have found that retinopathy induced by long-term exposure to hyperglycemia persists after glycemic control (8). Despite advances in the treatment with anti-angiogenic drugs (9), patients with DR might still experience adverse effects on their quality of life and financial circumstances. Accordingly, in order to reduce the prevalence of vision impairment and blindness caused by DR, it is imperative to discover more specific and sensitive biomarkers and explore its detailed etiology.

Early clinical characteristics of DR, such as microaneurysms and intraretinal microvascular abnormalities, are caused by changes in the cellular composition of the capillary wall (10). With the occurrence of capillary blockage and retinal ischemia in DR, neovascularization arises on the retina, resulting in proliferative DR (11). However, the pathological mechanism of DR, especially the formation mechanism of new blood vessels remains unclear. Thrombospondin-1 (*THBS1*) is a commonly matrix-cellular glycoprotein that plays a significant role in retinal vascular homeostasis (12, 13). Importantly, *THBS1* is a potent endogenous inhibitor of angiogenesis. *THBS1* was found to inhibit endothelial cell migration and proliferation and stimulate apoptosis by regulating vascular endothelial growth factor activity (14). *THBS1* may be involved in the development and progression of DR (15). Overexpression of *THBS1* impairs retinal vascular development and neointima formation in mice (16). In addition, deficiency of *THBS1* expression in retinal endothelial cells resulted in the accelerated proliferation and increased angiogenesis (16, 17). Moreover, we analyzed two gene expression datasets of DR patients (GSE94019 and GSE60436) (18, 19), which were downloaded from the Gene Expression Omnibus (GEO) (20), and found that the expression of *THBS1* was significantly up-regulated in the fibrovascular membrane of DR compared to normal retinal tissue in both datasets (adjusted P value = 3.58×10^-3^; 3.56×10^-8^) (data not published). Thus, we hypothesized that *THBS1* is a critical molecular in the development of DR, but the detailed mechanism underlying *THBS1* over-expression in DR is still unknown.

DNA methylation is a critical epigenetic alteration with the potential to illuminate the differential expression of *THBS1* in DR. DNA methylation status is closely correlated with gene expression (21). For example, hypermethylation of gene promoter regions can decreases DNA accessibility and inhibit transcription factors from binding, causing gene inactivation (i.e., inhibition of gene expression). Furthermore, since Maghbooli et al. first found higher global DNA methylation level in patients with DR (22), subsequent studies have confirmed that DNA methylation of some genes was indeed different in patients with DR (23–25). DNA methylation is therefore a critical factor related to gene expression, and cannot be ignored in the development of DR. According to our previous analysis of two datasets, differential expression of anti-angiogenic factor-*THBS1* was identified in patients with DR. However, whether *THBS1* methylation is involved in DR remains unknown. Moreover, the association between genetic variation and DNA methylation in humans has been extensively studied, and methylation quantitative trait loci (meQTL) may exert a regulatory effect on methylation at related cytosine-phosphate-guanine (CpG) sites (including cis and trans effect) (26–28). Particularly, meQTL pairs formed by SNPs and CpG loci tend to be enriched for functionally relevant features, such as gene expression, metabolic functions and clinical manifestations (29). Therefore, identifying the genetic regulation of DNA methylation can help us figure out the mechanisms by which genetic variation influences complex phenotypes. In light of these findings, exploring whether methylation of *THBS1* gene is genetically regulated may provide insight into the role of *THBS1* in DR and shed new light on the molecular networks and biological mechanisms of DR.

Our study aimed to determine whether overexpression of *THBS1* is associated with its promoter methylation level, and whether methylation of *THBS1* is regulated by genetic variants in DR.

## Methods and materials

### Study population

Patients diagnosed with DR and DM patients without retinal complications were recruited in the case-control study from communities in Shenzhen City, Guangdong Province, China. The patients recruited from 2019 to 2020 were for the study of *THBS1* methylation detection and specific SNPs genotyping, and the patients recruited from August to September 2022 were for *THBS1* mRNA testing. Patients in the case group were adult males or females with a history of T2DM, impaired vision, and previously diagnosed with DR by Fluorescence fundus angiography or optical coherence tomography. Patients in the control group were previously diagnosed with T2DM {according to the Chinese Guidelines for the Prevention and Treatment of Type 2 Diabetes [2017 Edition (30)]} without retinopathy. The case and control subjects were matched for age, sex, and duration of disease. Patients with diabetic foot, diabetic nephropathy, macular edema, history of other eye diseases or eye surgery, malignant tumors, severe liver and kidney dysfunction, mental illness, and alcohol or drug abuse were excluded. This study was approved by the Ethics Committee of Shenzhen Center for Chronic Disease Control. Written informed consent was signed by all participants.

### Questionnaire survey

Questionnaire survey was conducted among the patients recruited from 2019 to 2020 for demographic information (gender and age), anthropometric data [height, weight and body mass index (BMI)], diabetic duration, history of hypertension and information on lifestyle (smoking and drinking frequency) of patients enrolled. BMI = weight/height^2^ (kg/m^2^). Hypertension was defined as systolic blood pressure (SBP) ≥140 mmHg and/or diastolic blood pressure (DBP) ≥ 90 mmHg, or a prior history of hypertension or use of antihypertensive medications. Smokers are defined as those who smoke one or more cigarettes a day for more than one year. Drinkers were defined as those who consumed more than 50 ml alcohol per week on average and drank for more than six months. The collected questionnaire data was sorted out by using EPIDATA software and recorded by two people.

### Blood sample collection and DNA Extraction

5 ml fasting peripheral blood of all participants were collected by vacuum collection tubes after fasting for 12 hours. The blood samples of the first recruited patients were centrifuged, divided and stored in a refrigerator at -80℃. DNA was extracted from their peripheral blood by Blood Genomic DNA Extraction Kit (Omega, American). The blood samples of the secondly recruited patients were centrifuged, divided and used for subsequent mRNA level testing.

### Biochemical index detection

Glycosylated hemoglobin (HbA1c), fasting blood glucose (FPG), urea (UREA), creatinine (Cr), triglycerides (TG), total cholesterol (TC), low-density lipoprotein cholesterol (LDL-C) and high-density lipoprotein cholesterol (HDL-C) were tested among the patients recruited from 2019-2020 with Beckman-LX20 automatic biochemical analyzer.

### *THBS1* methylation detection

The detailed information on CpG islands and CpG sites of *THBS1* gene is provided in **Table S1**. Bisulfite conversion of DNA was performed by EZ DNA methylation-Gold ™ Kit (ZYMO, CA, USA). The modified DNA was amplified by multiple PCR (HotStart Taq polymerase, TaKaRa, Dalian, China). Primers with Index sequences were used to introduce specific tag sequences to the end of target and region by PCR amplification (Herculase^®^ II Fusion DNA Polymerase, Agilent Technologies, CA, USA). The information on Primer sequences of *THBS1* is provided in **Table S2**. The PCR products were electrophoresed with 2% agarose gel and purified with TIANGEN Gel Extraction kit (TIANGEN, Beijing, China). Finally, data on *THBS1* methylation was obtained using Illumina Hiseq or Nova SEQ platform for high-throughput sequencing.

### Genotyping of specific SNPs

Genomic DNA extracted from peripheral blood was analyzed by mass spectrometry using MassARRAY (Agena Bioscience), and then genotyping of those thirteen SNPs was performed. GTEx portal (https://www.gtexportal.org/home/index.html) was used to search for expression quantitative trait Loci (eQTL) of *THBS1* gene, and thirteen eQTLs existing both in whole blood and fibroblasts were selected as SNPs to be detected. Detailed information on SNPs is listed in **Table 1**.

**Table 1:** Detailed information about SNPs.

SNP	Chromosome	Location(GRCh38.p13)	Gene	Region	Alleles
rs11070177	15	39250064	C15orf54	2KB Upstream Variant	C>T
rs13329154	15	39252947	C15orf54	Non Coding Transcript Variant	C>T
rs143182940	15	39309678-39309680	–		delCTT
rs156657	15	39249331	C15orf54	2KB Upstream Variant	C>T
rs1847663	15	39253913	C15orf54	Non Coding Transcript Variant	A>G
rs201057385	15	39336247-39336252	–		delAAG
rs202208752	15	39321928-39321932	–		delGATAT
rs34401261	15	39248232-39248235	–		delTTC/dupTTC
rs34973764	15	39279332-39279333	–		insC/insG
rs36015436	15	39266666-39266668	–		delTA/dupTA
rs5812091	15	39304767	–		dupC
rs5812094	15	39313990-39313994	–		dupTGAC
rs71745389	15	39339794-39339798	–		delGAGA/dupGAGA

The genotyping process was completed by Shanghai Tianhao Biotechnology Company. The quality evaluation of a SNP included minor allele frequency (MAF>5%), call rate(>95%).

### mRNA extraction, cDNA synthesis and real time Quantitative PCR

Peripheral blood samples were treated with Red Blood Cell Lysis Buffer (Beyotime Biotechnology, Shanghai) and total RNA was extracted from cells using the Trizol method (TRIzol, Reagent life, USA). cDNA was synthesized from 1μg of total RNA using PrimeScriptTM RT reagent Kit with gDNA Eraser (Perfect real time) (TAKARA, Japan). The concentration and purity of RNA were determined by spectrophotometry (NanoVue Plus, GE healthcare UK limited, the United Kingdom). *THBS1* mRNA expression was measured by real time quantitative PCR(RT-PCR) with TB Green^®^ Premix Ex Taq™ II (TAKARA, Japan). The optimal number of PCR cycles and the mixing ratio of primers were determined according to the instruction of TB green reagent (Code No. RR820A). PCR products were quantified using TB Green. Beta-actin (ACTB) was used as an endogenous control to normalize expression levels. The ranges of linear amplification for the target gene and for the ACTB genes were studied. Data were normalized relative to the expression level of ACTB for each sample. Primers used for RT-PCR were human *THBS1*-1, 5’-TTGTCTTTGGAACCACACCA-3’(sense) and 5’-TGGACAGCTCATCACAGGAG-3’ (antisense); and ACTB 5’-GATGAGATTGGCATGGCTTT-3’ (Sense) and 5’-CACCTTCACCGGTCAGTTT -3’ (antisense). The expression levels of mRNA were represented as 2^-ΔΔCT^.

### Statistic analysis

In terms of basic characteristics, continuous variables with normal distribution were presented as mean ± standard deviation, and independent two-sample t-tests were used to compare differences between the case and control group. Continuous variables without normal distribution were presented as medians (percentile 25, percentile 75), Wilcoxon rank sum test was performed to identify differences between groups. Categorical variables were presented using percentages and chi-square tests were used to compare differences between groups. All those tests were conducted two-tailed in IBM SPSS 25 software, and P<0.05 was considered significant.

The differentially methylated CpG loci of *THBS1* were identified using univariate logistic regression in SPSS software. Associations between each SNP and methylation levels of differentially methylated CpG loci were tested to identify meQTLs using linear regression in the software PLINK (31). The independent variable was the genotype of the SNP, and the additive model was adopted. Given that we had previously tried to keep the distribution of most critical confounding factors- age, gender and diabetic duration to be the same between the case group and the control group, so we did not adjust for other factors. Cis-meQTL was defined as being less than 500kb upstream or downstream of CpG sites from the associated CpG loci, otherwise as trans-meQTLs. To examine the independence of these meQTLs, linkage disequilibrium (LD) analysis was performed by Haploview 4.2 programme (32). r^2^ was obtained as a measure of LD based on our study population. To explore the relationship between meQTLs and DR, binary logistic regressions were performed in dominant, recessive and additive models in SPSS software. HbA1c and TC were adjusted in three models. P<0.05 was considered significant.

To determine whether methylation at CpG loci was an intermediate factor between genetic variation and DR, we performed a mediation analysis by using macro PROCESS (v3.4 by Andrew F. Hayes) in SPSS. Model 4 was used for our analysis, which represented a simple mediation model (the type occurring when only one variable regulated the effect of a cause on an outcome) (33). 5000 bias-corrected Bootstrap sample was used for the significance test. It is sufficient to support a claim of mediation effect when the 95% bootstrap confidence intervals of the indirect effect (as quantified with ab) does not include zero (34). We can determine whether there is an intermediate effect of methylation, by calculating whether the indirect effect of genetic variation on disease is significant.

## Results

### Characteristics of the participants

From 2019 to 2020, 150 patients diagnosed with DR and 150 DM patients without retinal complications were enrolled in our study. Their clinical characteristics are summarized in **Table 2**. Since we matched the age, sex and duration of disease between the case group and the control group, the mean age of both groups was about 56 years, 55% were female, and the median duration of disease was 8 and 9 years, respectively. As illustrated in the table, BMI and the proportion of hypertension patients were comparable between the case and control groups. HbA1c, FPG, UREA, TC, LDL-C in the case group were significantly higher than those in the control group (P value<0.05).

**Table 2:** Clinical characteristics of subjects.

	Cases(n=150)	Controls(n=150)	P value
Age,years	56.63 ± 9.29	56.69 ± 9.07	0.955
Female,n(%)	55(36.7)	55(36.7)	1
BMI,kg/m^2^	24.61 ± 3.04	24.86 ± 3.11	0.502
Diabetic duration,years	8.00(4.00,13.00)	9.00(4.50,12,5)	0.593
Hypertension,n(%)	73(48.7)	79(42.7)	0.564
Smoking,n(%)	40(26.7)	35(23.3)	0.594
Drinking,n(%)	52(34.7)	45(30.0)	0.459
HbA1c,%	7.10(6.13,8.50)	6.60(5.80,7.30)	0.002
FPG,mmol/L	8.35(6.70,10.80)	6.90(5.85,8.65)	<0.001
UREA,mmol/L	5.30(4.43,6.55)	4.90(4.20,5.90)	0.021
Cr,umol/L	71.80(57.10,85.10)	72.30(58.65,88.10)	0.542
TG,mmol/L	1.58(1.13,2.25)	1.54(1.03,2.30)	0.489
TC,mmol/L	5.33 ± 1.34	4.95 ± 1.17	0.008
LDL-C,mmol/L	3.62 ± 0.92	3.33 ± 0.87	0.009
HDL-C,mmol/L	1.28 ± 0.29	1.24 ± 0.27	0.226

HbA1c, Glycosylated hemoglobin; FPG, fasting blood glucose; UREA, urea; Cr, creatinine; TG, triglycerides; TC, total cholesterol; LDL-C, low density lipoprotein cholesterol; HDL-C, high-density lipoprotein cholesterol.

From August to September 2022, seven DR patients and seven DM patients without retinal complications were enrolled in our study. Since we had matched age and gender of the participants in the case group and control group, the average ages of the case group and the control group are 60.14 ± 8.17 and 59.86 ± 7.51, with four females in each group.

### Methylation analysis

**Figure 1:**
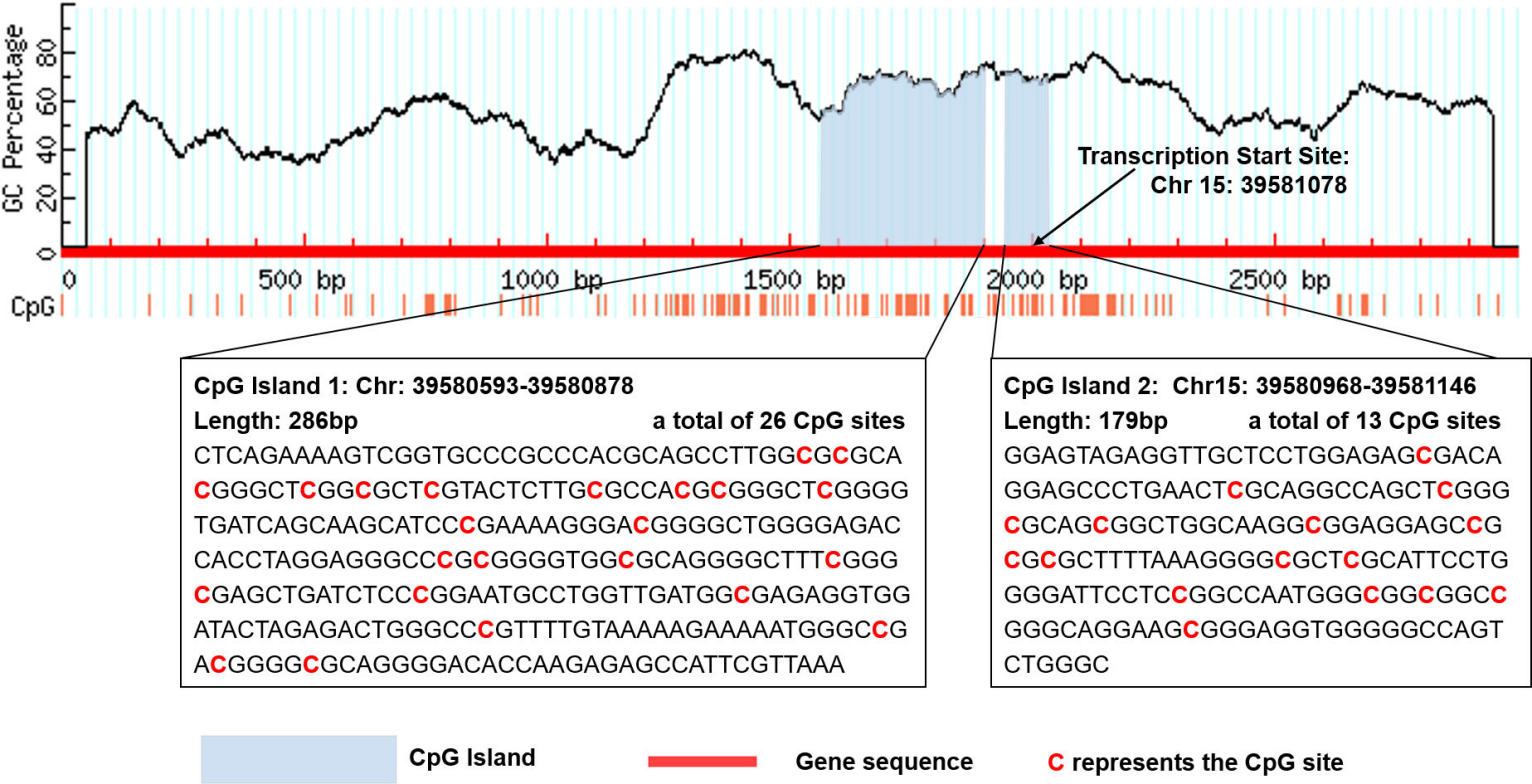
Location of the two CpG islands in THBS1 gene.

**Figure 2:**
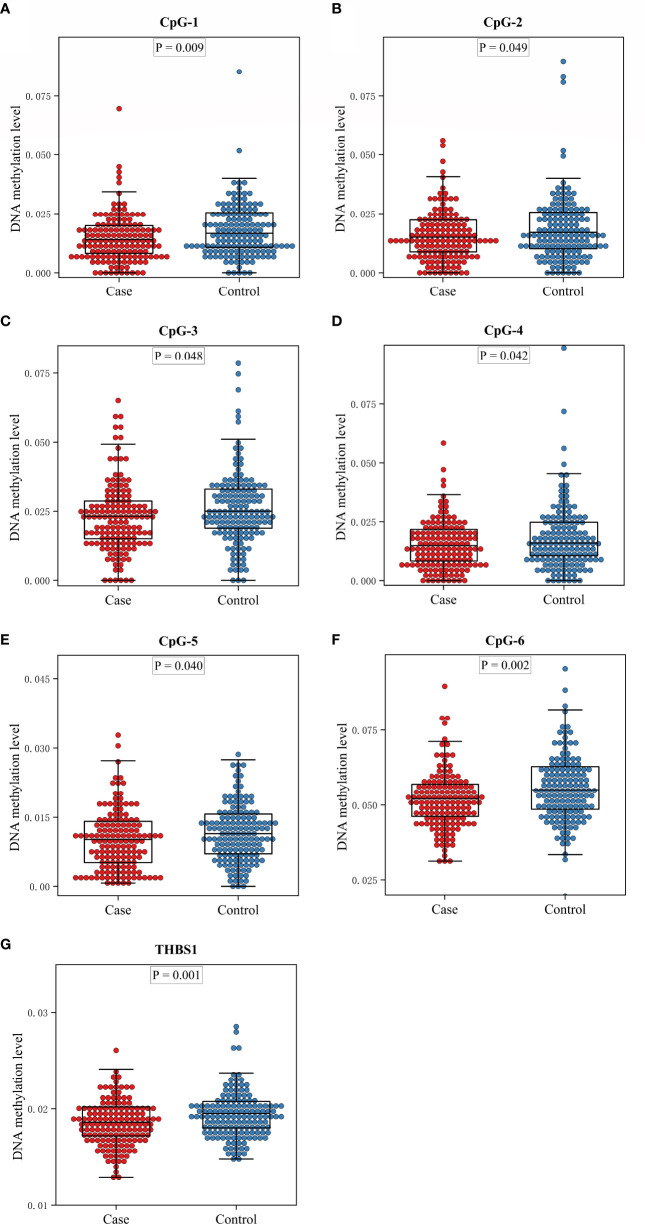
The box plots of methylation levels of *THBS1* in the case and control groups. **(A–F)** Methylation levels of six CpG loci with significant difference in the case and control group. **(G)** Methylation levels of THBS1 in the case and control group.

There are two CpG islands (CGIs) in *THBS1* gene, which contains 23 and 16 CpG sites respectively, and five CpG sites of CGI-2 are in the gene body of *THBS1* **(** **Figure 1** **)**. Methylation levels of 39 CpG loci on *THBS1* gene were tested in all subjects (**Table S1**). The patients with DR showed a lower methylation level of *THBS1* than the patients with DM (P value = 0.002) (**Table S1**). To be specific, the methylation levels of six CpG loci in the patients with DR were significantly different from those with DM, and all the 6 CpG loci in the patients with DR showed hypomethylation (P value<0.05) (**Table 3**). All these six sites are located in the promoter region of *THBS1*, CpG-5 locates downstream of the transcription start site (TSS), and other sites locates upstream of the TSS. The box plots of methylation levels of *THBS1* and six CpG loci in the two groups are shown in **Figure 2**.

**Table 3:** Methylation analysis of *THBS1*.

	Chromosome 15 position(hg38)	Distance to TSS*	Menthylation Level, %		OR (95%CI)	P-value
			Cases	Controls		
CpG-1	39580843	-235	1.5140 ± 1.0131	1.8454 ± 1.1113	0.733 (0.581-0.926)	0.009
CpG-2	39580809	-269	1.6157 ± 1.0592	1.9062 ± 1.4094	0.823 (0.678-0.999)	0.049
CpG-3	39580741	-337	2.3637 ± 1.2478	2.6636 ± 1.3417	0.834 (0.697-0.999)	0.048
CpG-4	39580731	-347	1.5805 ± 1.0157	1.8695 ± 1.3641	0.812 (0.663-0.993)	0.042
CpG-5	39581083	5	1.0382 ± 0.6342	1.1875 ± 0.6098	0.678 (0.468-0.982)	0.040
CpG-6	39581002	-76	5.2338 ± 0.9362	5.6450 ± 1.1914	0.687 (0.545-0.866)	0.002
***THBS1***	39581078-39599466	–	1.8701 ± 0.2300	1.9704 ± 0.2923	0.191 (0.069-0.532)	0.002

*The distance of the site to the transcription start site on the reference genome, with a minus sign indicating that the site is upstream of the transcription start site.

### Identification of *THBS1* meQTL

**Figure 3:**
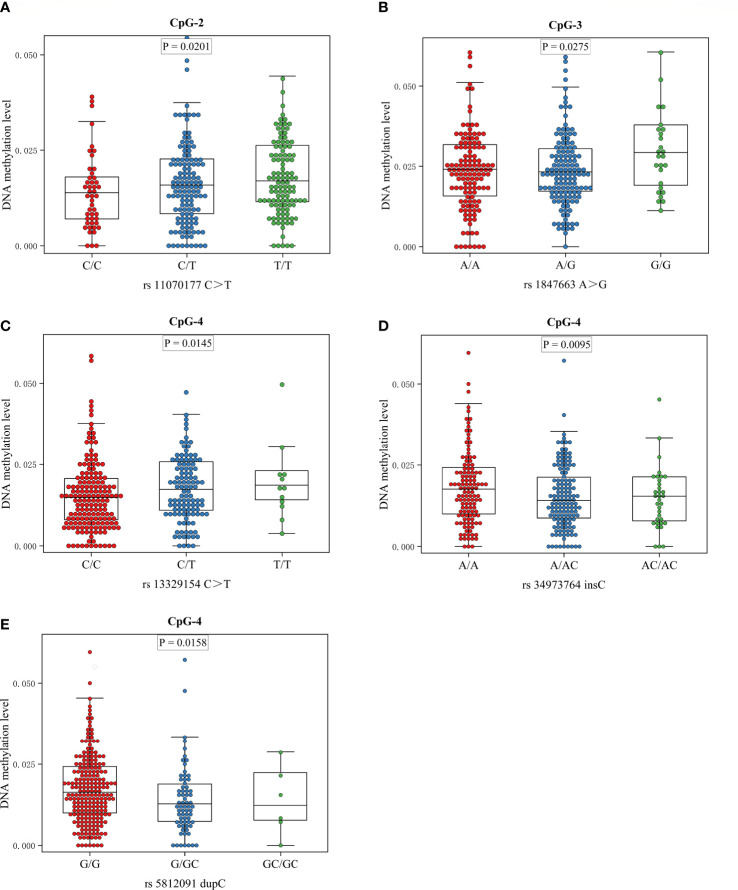
Boxplots showing methylation level plotted against genotypes for five meQTLs: Center lines show the medians; box limits indicate the 25th and 75th percentiles; whiskers extend to the 5th and 95th percentiles. **(A)** The association between genotype at rs11070177 and methylation level at CpG-2; **(B)** the association between genotype at rs1847663 and methylation level at CpG-3; **(C–E)** the association between genotypes at rs13329154, rs34973764, rs5812091 and methylation level at CpG-4.

**Figure 4:**
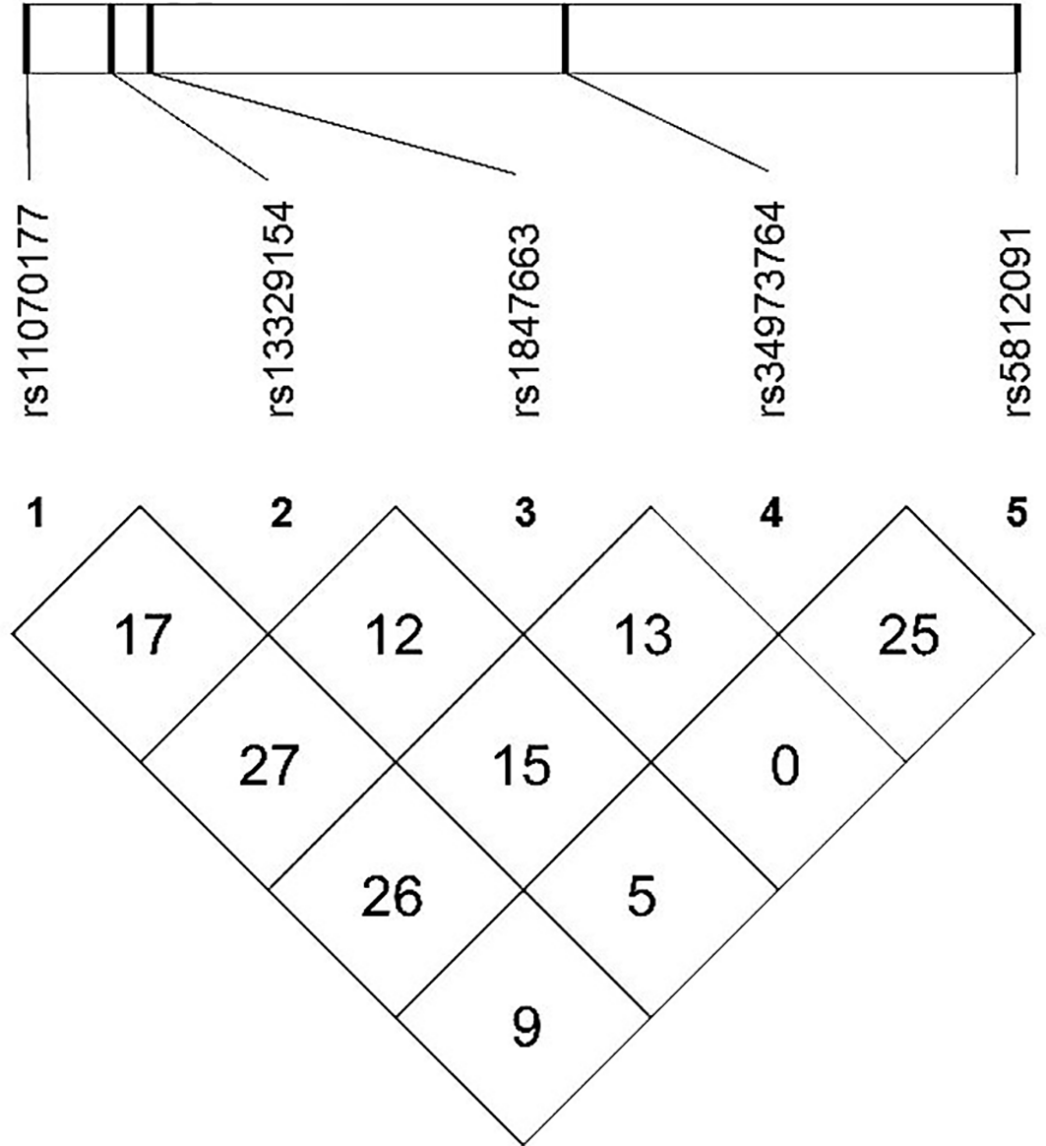
Linkage disequilibrium (LD) map among five meQTLs: LD r^2^(%) based on our study population are shown in the boxes.

The meQTL association analyses between 6 differentially methylated CpG sites and 13 SNPS were performed by the software PLINK, and the results are shown in **Table S3**. Five SNPs were associated with three differentially methylated CpG loci, all of which were cis-meQTLs upstream of the associated CpG loci. Box plots of methylation level plotted against genotypes for five meQTLs are shown in **Figure 3**. rs13329154(C>T), rs34973764(insC), rs5812091(dupC) were cis-meQTLs of CpG-4 (P value=0.0145, 0.0095, 0.0158; distance between SNPs and associated CpG site: 319kb, 297kb, 269kb respectively). Variations in these SNPs were associated with reduced methylation level. In addition, rs11070177(C>T) and rs1847663(A>G) were cis-meQTLs of CpG-2 and CpG-3 (P value=0.0201, 0.0275; distance between SNPs and associated CpG site: 323kb, 319kb), and methylation levels of CpG-2 and CpG-3 increased with the number of allelic mutations. LD analysis between these five meQTLs showed a low degree of LD (r ^2^<30%) (**Figure 4**). Association analysis between these five meQTLs and DR showed that none of these meQTLs were associated with DR in dominant, recessive and additive models **(** **Table S4** **)**.

### Mediation effect

**Figure 5:**
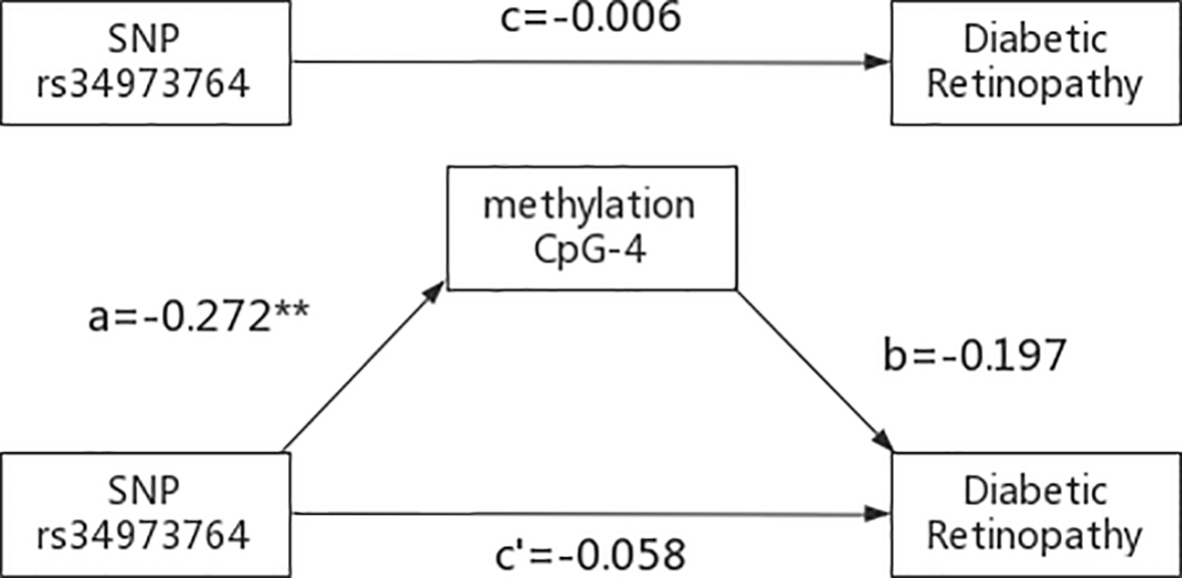
Results of the mediation analysis: Indirect effect of rs34973764 on diabetic retinopathy through methylation of CpG-4 and total effect (regression coefficients). **p ≤.001.

The indirect mediation routes of five meQTLs on DR through methylation of three CpG loci are shown in **Table 4**. In the five established mediation models, CpG-4 methylation significantly mediated the effect of the polymorphism rs34973764 on DR (B=0.0535, Boot 95%CI: 0.004~0.1336) **(** **Table 4** **)**. Analysis of indirect effects showed that the variant allele of rs34973764 predicted hypomethylation levels at CpG-4 (a=-0.272, p value=0.009), and the incidence of DR increased with hypomethylation level at CpG-4 (b=-0.197, p value=0.0596) **(** **Figure 5** **)**. Although the total and direct effect of rs34973764 on DR were not significant (total effect: c=-0.006, p value=0.973; direct effect: c’=-.0575, p value=0.7464), the indirect effect showed significant. Therefore, rs34973764 may act as a risk SNP for DR through CpG-4.

**Table 4:** The results of mediation analysis.

Indirect effect					B	BootSE	Boot 95%CI
rs11070177	→	CpG-2	→	DR	-0.0488	0.0328	-0.1254~0.0003
rs1847663	→	CpG-3	→	DR	-0.0438	0.0322	-0.1180~0.0092
rs13329154	→	CpG-4	→	DR	-0.0547	0.0433	-0.1586~0.004
rs34973764^*^	→	CpG-4	→	DR	0.0535	0.0349	0.0004~0.1336
rs5812091	→	CpG-4	→	DR	0.0635	0.0453	-0.0047~0.1727

^*^Bootstrap 95% confidence interval of the indirect effect does not include zero.

### *THBS1* mRNA expression

*THBS1* mRNA expression in peripheral blood was significantly higher in DR patients than in DM patients (2^-ΔΔCT^ median (percentile 25, 75): 3.67(1.41-8.03) vs. 1.00(0.53-1.33), p value=0.025). Comparison of *THBS1* mRNA expression levels in the two groups is shown in **Figure S1**.

## Discussion

In our study, the patients with DR showed significant hypomethylation of *THBS1* compared to those with T2DM, and 6 out of 39 CpG sites showed hypomethylation. Moreover, 5 out of 13 SNPs were identified to be associated with three differentially methylated CpG loci of *THBS1*. CpG-4 methylation mediated the effect of rs34973764 on DR in the mediation analysis, providing data evidence that *THBS1* methylation may be regulated by genetic variation in DR.

*THBS1* is an endogenous molecule functioning as anti-angiogenesis and has been proven to regulate ocular vascular homeostasis (35). Numerous studies revealed the critical role of *THBS1* in the onset and progression of DR, especially in its neovascularization and vascular abnormalities. In the differential expression analysis performed prior to this study, results showed that the mRNA expression of *THBS1* in the fibrovascular membrane of DR patients was significantly up-regulated in both datasets (GSE94019 and GSE60436) (18, 19). This result has been confirmed in other studies. Bian et al. found that patients with DR exhibited significantly elevated serum THBS1 compared to patients with T2DM, patients with proliferative DR in particular showed the highest *THBS1* (36). Wang et al. observed high expression of *THBS1* in the retina of DR rats (37). Wu et al. identified that mice with *THBS1* over-expressing in the lens showed attenuated retinal vascular development and impaired neovascularization (16). Hence, the expression of THBS1 is highly associated with the formation of new blood vessels, which may have a significant impact on the development of DR.

It is widely accepted that the expression of genes is affected by genetic regulation or epigenetic modification, which is adjustable and susceptible to environmental factors. So, it is imperative to figure out the association between the expression and epigenetic modification of *THBS1* in DR. Given that DNA methylation is an important epigenetic modification that has been studied the most, our study focused on how DNA methylation affects expression in DR. In our research, the methylation level of *THBS1* was significantly lower in the patients with DR compared to with DM. Besides, methylation levels of six CpG sites of *THBS1* showed a significant difference between those two groups, and all of these sites showed decreased DNA methylation in cases with DR. Then we examined *THBS1* mRNA expression levels in another recruited patients, and the results showed that the mRNA expression level of *THBS1* in peripheral blood was significantly higher in DR patients than in DM patients. In addition, patients with DR are typically poor in glycemic control, as supported by our data. Studies confirmed that hyperglycemia can result in irreversible alterations in the activity of DNA methyltransferases and hydroxymethylase (38). Moreover, *THBS1* expression was significantly increased in the high-glucose environment in keratinocytes and diabetic rat model, which was induced through DNA hypomethylation (39). Thus, we speculate that high glucose may have an impact on the *THBS1* methylation *via* enzyme activity, resulting in alterations of *THBS1* expression in DR. Since the existing research is restricted, additional research is necessary to clarify the mechanism of methylation and expression of *THBS1* in DR.DNA methylation has been previously described to have a genetic basis, and numerous studies have investigated the relationship between DNA methylation and genetic variation throughout the genome to identify meQTLs (40). In our meQTL analysis, there were five cis-CpG-SNPs associations identified, including three *THBS1* methylation loci and five SNPs. The rare allele of rs13329154, rs34973764, rs5812091 exhibited significantly decreased levels of CpG-4 methylation, while the rare allele of rs11070177 and rs1847663 exhibited significantly increased levels of CpG-2 and CpG-3 methylation respectively. Rs13329154, rs11070177 and rs1847663 are located in the noncoding region of C15orf54 gene, while rs34973764, rs5812091 have not been characterized. C15orf54 gene has not been well understood, and up-regulated expression of C15orf54 was first found to be associated with the risk of gastric cancer in a recent bioinformatics analysis (41). In addition, there is no association between these five meQTLs and DR in dominant, recessive and additive models in our research, and these five meQTLs have not been reported to be associated to any phenotypes or diseases in prior studies. Hence, more research is needed to determine how C15orf54 gene polymorphism affects *THBS1* methylation in DR.

meQTLs are highly enriched in GWAS signals (42), implying that meQTLs are associated with an elevated risk of disease. Studies have shown that meQTLs may exert an influence on disease risk *via* altering DNA methylation (40). In the mediation study, CpG-4 methylation mediated the effect of rs34973764 on DR, which may be supporting evidence for genetic regulation of DNA methylation in DR. Although the total effect of rs34973764 on DR was not significant, rs34973764 had a significant indirect effect on the DR *via* the mediator of CpG-4 methylation. One possible explanation is that rs34973764 functions in DR *via* two or more additional mediation pathways, and those additional pathways operate oppositely from the CpG-4 methylation pathway. However, the function of rs34973764 and its impact on CPG-4 are not yet fully understood, so the underlying molecular processes of meQTLs are unknown. The most common explanation for the cis-meQTL effect is that SNPs at protein binding sites affect the function of sequence-specific binding proteins, such as transcription factors, and thus change the methylation pattern of adjacent CpGs (40). Future research combining SNPs, DNA methylation, and gene expression are needed to better understand the pathology of DR.

There are several limitations in our study. Firstly, DR is classified as either proliferative or non-proliferative (11), but in our study, we did not divide patients into those two categories. Therefore, we could not obtain dynamic changes in *THBS1* methylation level as the disease progressed. Secondly, the SNPs we detected were not from genome-wide SNPs, but eQTLs related to *THBS1*, which represented only a small fraction of meQTLs of *THBS1*. Thirdly, the sample size was relatively insufficient, especially in the detection of *THBS1* mRNA expression levels, hence case-control and cohort studies with larger sample size are needed to confirm these findings.

In conclusion, *THBS1* overexpression is related to *THBS1* hypomethylation in patients with DR. DNA methylation may be genetically controlled in DR.

## Data availability statement

The original contributions presented in the study are included in the article/**Supplementary Material**. Further inquiries can be directed to the corresponding authors.

## Ethics statement

The studies involving human participants were reviewed and approved by the Ethics Committee of Shenzhen Center for Chronic Disease Control. The patients/participants provided their written informed consent to participate in this study.

## Author contributions

YL and CG contributed equally to this work. CG and WH contributed to the design of the work. YL contributed to manuscript written and statistical analyses. Data acquisition and analysis were carried out by YX and XL. JY and CG reviewed the paper. All authors contributed to the article and approved the submitted version.

## Funding

This work was supported by the Sanming Project of Medicine of Shenzhen (SZSM201811057), National Natural Science of Guang dong province (2019A1515010358) and Guangdong Medical Research Fund Project (2017196).

## Acknowledgments

We thank all participants for providing samples and those involved in the sample collection.

## Conflict of interest

The authors declare that the research was conducted in the absence of any commercial or financial relationships that could be construed as a potential conflict of interest.

## Publisher’s note

All claims expressed in this article are solely those of the authors and do not necessarily represent those of their affiliated organizations, or those of the publisher, the editors and the reviewers. Any product that may be evaluated in this article, or claim that may be made by its manufacturer, is not guaranteed or endorsed by the publisher.
