# Supplementary material for: Genetic regulation of THBS1 methylation in diabetic retinopathy
Source: Front Endocrinol (Lausanne). 2022 Nov 14;13:991803. doi: 10.3389/fendo.2022.991803 (PMC9702561; doi:10.3389/fendo.2022.991803)
Supplement: Supplementary file 1 [file DataSheet_1.pdf]

Supplementary Material

Table S1. detailed information of CpG sites of THBS1 gene and results of methylation analysis

CpG site number	Methylation island	Position[1]	Chromsome	Genome Position	Distance to TSS[2]	Type	Menthylation Level, %		OR (95%CI)	P-value
							Cases	Controls		
1	THBS1 CGI-1	36	15	39580843	-235	CG	0.0151 ± 0.0101	0.0185 ± 0.0111	0.7334(0.5809-0.926)	**0.009**
2	THBS1 CGI-1	38	15	39580841	-237	CG	0.0169 ± 0.0108	0.0173 ± 0.0103	0.9705(0.7825-1.2037)	0.785
3	THBS1 CGI-1	42	15	39580837	-241	CG	0.0119 ± 0.0093	0.0139 ± 0.0102	0.8027(0.6267-1.0281)	0.082
4	THBS1 CGI-1	48	15	39580831	-247	CG	0.0115 ± 0.0082	0.0106 ± 0.0075	1.1543(0.8644-1.5414)	0.331
5	THBS1 CGI-1	51	15	39580828	-250	CG	0.0161 ± 0.0095	0.0175 ± 0.0111	0.8805(0.7045-1.1005)	0.263
6	THBS1 CGI-1	55	15	39580824	-254	CG	0.0135 ± 0.0093	0.0149 ± 0.0095	0.8502(0.6654-1.0865)	0.195
7	THBS1 CGI-1	65	15	39580814	-264	CG	0.0177 ± 0.0121	0.0180 ± 0.0113	0.9828(0.8094-1.1932)	0.861
8	THBS1 CGI-1	70	15	39580809	-269	CG	0.0162 ± 0.0106	0.0191 ± 0.0141	0.8229(0.6778-0.9991)	**0.049**
9	THBS1 CGI-1	72	15	39580807	-271	CG	0.0146 ± 0.0099	0.0164 ± 0.0117	0.8519(0.686-1.0579)	0.147
10	THBS1 CGI-1	78	15	39580801	-277	CG	0.0303 ± 0.0140	0.0317 ± 0.0159	0.941(0.8079-1.0961)	0.435
11	THBS1 CGI-1	99	15	39580780	-298	CG	0.0161 ± 0.0116	0.0177 ± 0.0120	0.8926(0.7343-1.085)	0.254

12	THBS1 CGI-1	109	15	39580770	-308	CG	0.0469 ± 0.0196	0.0478 ± 0.0191	0.9758(0.8676-1.0976)	0.683
13	THBS1 CGI-1	138	15	39580741	-337	CG	0.0236 ± 0.0125	0.0266 ± 0.0134	0.834(0.6967-0.9985)	**0.048**
14	THBS1 CGI-1	140	15	39580739	-339	CG	0.0417 ± 0.0163	0.0426 ± 0.0149	0.9624(0.8319-1.1133)	0.606
15	THBS1 CGI-1	148	15	39580731	-347	CG	0.0158 ± 0.0102	0.0187 ± 0.0136	0.8115(0.6633-0.9928)	**0.042**
16	THBS1 CGI-1	160	15	39580719	-359	CG	0.0135 ± 0.0093	0.0141 ± 0.0098	0.9335(0.7358-1.1842)	0.571
17	THBS1 CGI-1	164	15	39580715	-363	CG	0.0082 ± 0.0069	0.0089 ± 0.0080	0.8846(0.6518-1.2006)	0.431
18	THBS1 CGI-1	177	15	39580702	-376	CG	0.0118 ± 0.0090	0.0123 ±0.0097	0.9538(0.7482-1.2159)	0.703
19	THBS1 CGI-1	196	15	39580683	-395	CG	0.0104 ± 0.0085	0.0111 ± 0.0083	0.9155(0.6991-1.1989)	0.521
20	THBS1 CGI-1	223	15	39580656	-422	CG	0.0134 ± 0.0088	0.0131 ± 0.0093	1.0369(0.8075-1.3313)	0.777
21	THBS1 CGI-1	247	15	39580632	-446	CG	0.0183 ± 0.0134	0.0183 ± 0.0118	0.9995(0.8349-1.1966)	0.996
22	THBS1 CGI-1	250	15	39580629	-449	CG	0.0172 ± 0.0133	0.0174 ± 0.0128	0.988(0.8303-1.1758)	0.892
23	THBS1 CGI-1	255	15	39580624	-454	CG	0.0250 ± 0.0135	0.0275 ± 0.0130	0.8655(0.7277-1.0294)	0.103
24	THBS1 CGI-2	25	15	39581122	44	CG	0.0255 ± 0.0068	0.0256 ± 0.0088	0.9841(0.7369-1.3143)	0.914
25	THBS1 CGI-2	43	15	39581104	26	CG	0.0127 ± 0.0040	0.0131 ± 0.0048	0.8173(0.489-1.3662)	0.442
26	THBS1 CGI-2	55	15	39581092	14	CG	0.0107 ± 0.0041	0.0121 ± 0.0075	0.6306(0.3946-1.0078)	0.054

27	THBS1 CGI-2	59	15	39581088	10	CG	0.0157 ± 0.0048	0.0154 ± 0.0052	1.142(0.7258-1.7968)	0.566
28	THBS1 CGI-2	64	15	39581083	5	CG	0.0104 ± 0.0063	0.0119 ± 0.0061	0.6776(0.4676-0.9821)	**0.040**
29	THBS1 CGI-2	76	15	39581071	-7	CG	0.0290 ± 0.0090	0.0298 ± 0.0102	0.9164(0.7229-1.1616)	0.470
30	THBS1 CGI-2	85	15	39581062	-16	CG	0.0196 ± 0.0059	0.0192 ± 0.0058	1.1193(0.7578-1.6532)	0.571
31	THBS1 CGI-2	87	15	39581060	-18	CG	0.0146 ± 0.0049	0.0149 ± 0.0054	0.8937(0.5753-1.3885)	0.617
32	THBS1 CGI-2	89	15	39581058	-20	CG	0.0206 ± 0.0062	0.0207 ± 0.0063	0.9842(0.6845-1.4151)	0.931
33	THBS1 CGI-2	103	15	39581044	-34	CG	0.0173 ± 0.0055	0.0166 ± 0.0058	1.2455(0.8315-1.8656)	0.287
34	THBS1 CGI-2	107	15	39581040	-38	CG	0.0151 ± 0.0048	0.0158 ± 0.0058	0.7975(0.5174-1.2293)	0.305
35	THBS1 CGI-2	127	15	39581020	-58	CG	0.0161 ± 0.0045	0.0167 ± 0.0059	0.8001(0.5178-1.2364)	0.315
36	THBS1 CGI-2	138	15	39581009	-69	CG	0.0113 ± 0.0041	0.0122 ± 0.0058	0.6831(0.4184-1.1152)	0.127
37	THBS1 CGI-2	141	15	39581006	-72	CG	0.0119 ± 0.0041	0.0124 ± 0.0044	0.7548(0.4418-1.2896)	0.303
38	THBS1 CGI-2	145	15	39581002	-76	CG	0.0523 ± 0.0094	0.0564 ± 0.0119	0.6871(0.5451-0.8661)	**0.002**
39	THBS1 CGI-2	156	15	39580991	-87	CG	0.0208 ± 0.0052	0.0221 ± 0.0066	0.6907(0.4689-1.0174)	0.061
**THBS1**	-	-	15	39581078-39599466	-	-	0.0187±0.0230	0.0197±0.0029	0.191 (0.069-0.532)	**0.002**

CGI CpG island; [1]The position of the methylation site on the CGI; [2]The distance of the site to the transcription start site on the reference genome, with a minus sign indicating that the site is upstream of the transcription start site

Table S2. primer sequences of THBS1

CpG Island	Chr	Gene	Length	Primer-Forward	Primer-Reverse	Target Sequence
THBS CGI-1	15	THBS1	286	TTTAGAAA<br>AGTYGGTG<br>TTYGTTTA<br>YGTAGT	TTTAA<br>CRAAT<br>AACTC<br>TCTTA<br>ATATC<br>CCCTA<br>C	CTCAGAAAAGTCGGTGCCCGCCCACGCAGCCTTGG**C**G**C**GCA**C**<br>GGGCT**C**GG**C**GCT**C**GTACTCTTG**C**GCCA**C**G**C**GGGCT**C**GGGGTG<br>ATCAGCAAGCATCC**C**GAAAAGGGA**C**GGGGCTGGGGAGACCAC<br>CTAGGAGGGCC**C**G**C**GGGGTGG**C**GCAGGGGCTTT**C**GGG**C**GAGC<br>TGATCTCC**C**GGAATGCCTGGTTGATGG**C**GAGAGGTGGATACTA<br>GAGACTGGGCC**C**GTTTTGTAAAAAGAAAAATGGGC**C**GA**C**GGG<br>G**C**GCAGGGGACACCAAGAGAGCCATTCGTTAAA
THBS CGI-2	15	THBS1	179	GGAGTAG<br>AGGTTGTT<br>TTTGGAGA	ACCCA<br>AACTA<br>ACCCC<br>CACCT	GGAGTAGAGGTTGCTCCTGGAGAG**C**GACAGGAGCCCTGAACT<br>**C**GCAGGCCAGCT**C**GGG**C**GCAG**C**GGCTGGCAAGG**C**GGAGGAGC<br>**C**G**C**G**C**GCTTTTAAAGGGG**C**GCT**C**GCATTCCTGGGGATTCCTC**C**<br>GGCCAATGGG**C**GG**C**GGC**C**GGGCAGGAAG**C**GGGAGGTGGGGGC<br>CAGTCTGGGC

CGI CpG island; Chr chromosome

Table S3. meQTL analysis of differentially methylated CpG sites

CpG(chr:position(hg38))	SNP	chromosome	SNP position(hg38)	P
CpG-1(15:39580843)	rs34401261	15	39248234	0.9174
	rs156657	15	39249331	0.9544
	rs11070177	15	39250064	0.4942
	rs13329154	15	39252947	0.5382
	rs1847663	15	39253913	0.6062
	rs36015436	15	39266667	0.6186
	rs34973764	15	39279332	0.7838
	rs5812091	15	39304767	0.2241
	rs143182940	15	39309679	0.3255
	rs5812094	15	39313992	0.3678
	rs202208752	15	39321930	0.1793
	rs201057385	15	39336250	0.4417
	rs71745389	15	39339796	0.6493
CpG-2(15:39580809)	rs34401261	15	39248234	0.06954
	rs156657	15	39249331	0.05695
	rs11070177	15	39250064	**0.0201**
	rs13329154	15	39252947	0.6793
	rs1847663	15	39253913	0.07011
	rs36015436	15	39266667	0.8393
	rs34973764	15	39279332	0.4584
	rs5812091	15	39304767	0.07613
	rs143182940	15	39309679	0.4586
	rs5812094	15	39313992	0.09555
	rs202208752	15	39321930	0.2812
	rs201057385	15	39336250	0.1677
	rs71745389	15	39339796	0.8177
CpG-3(15:39580741)	rs34401261	15	39248234	0.225
	rs156657	15	39249331	0.2236

	rs11070177	15	39250064	0.3354
	rs13329154	15	39252947	0.2349
	rs1847663	15	39253913	**0.0275**
	rs36015436	15	39266667	0.9472
	rs34973764	15	39279332	0.816
	rs5812091	15	39304767	0.1859
	rs143182940	15	39309679	0.8827
	rs5812094	15	39313992	0.4007
	rs202208752	15	39321930	0.4696
	rs201057385	15	39336250	0.4856
	rs71745389	15	39339796	0.1479
CpG-4(15:39580731)	rs34401261	15	39248234	0.06036
	rs156657	15	39249331	0.06997
	rs11070177	15	39250064	0.4201
	rs13329154	15	39252947	**0.0145**
	rs1847663	15	39253913	0.472
	rs36015436	15	39266667	0.0914
	rs34973764	15	39279332	**0.0095**
	rs5812091	15	39304767	**0.0158**
	rs143182940	15	39309679	0.1034
	rs5812094	15	39313992	0.2028
	rs202208752	15	39321930	0.1497
	rs201057385	15	39336250	0.1746
	rs71745389	15	39339796	0.5376
CpG-5(15:39581083)	rs34401261	15	39248234	0.6565
	rs156657	15	39249331	0.745
	rs11070177	15	39250064	0.759
	rs13329154	15	39252947	0.4903
	rs1847663	15	39253913	0.854
	rs36015436	15	39266667	0.8407

	rs34973764	15	39279332	0.7034
	rs5812091	15	39304767	0.1811
	rs143182940	15	39309679	0.5046
	rs5812094	15	39313992	0.27
	rs202208752	15	39321930	0.3526
	rs201057385	15	39336250	0.3171
	rs71745389	15	39339796	0.558
CpG-6(15:39581002)	rs34401261	15	39248234	0.9088
	rs156657	15	39249331	0.9093
	rs11070177	15	39250064	0.8558
	rs13329154	15	39252947	0.77
	rs1847663	15	39253913	0.933
	rs36015436	15	39266667	0.7924
	rs34973764	15	39279332	0.9252
	rs5812091	15	39304767	0.5427
	rs143182940	15	39309679	0.4397
	rs5812094	15	39313992	0.9445
	rs202208752	15	39321930	0.3767
	rs201057385	15	39336250	0.5949
	rs71745389	15	39339796	0.8207

Table S4. Association of meQTLs with DR

SNP	Genotype	case group	control group	Dominant Model		Recessive Model		Addictive Model	
				OR (95%CI)	P-value	OR (95%CI)	P-value	OR (95%CI)	P-value
rs11070177 (C>T)	C/C	28	24	1.190 (0.625-2.268)	0.596	1.182 (0.728-1.916)	0.499	0.881 (0.631-1.231)	0.459
	C/T	78	80						
	T/T	68	70						
rs13329154 (C>T)	C/C	102	101	1.262 (0.781-2.041)	0.342	1.080 (0.329-3.553)	0.899	0.836 (0.554-1.261)	0.392
	C/T	65	67						
	T/T	7	7						
rs1847663 (A>G)	A/A	76	76	1.076 (0.670-1.728)	0.762	0.896 (0.402-1.996)	0.788	0.980 (0.681-1.410)	0.912
	A/G	82	84						
	G/G	16	14						
rs34973764 (insC/insG)	A/A	77	68	1.205 (0.749-1.939)	0.443	0.701 (0.332-1.481)	0.352	0.978 (0.687-1.391)	0.901
	A/AC	73	87						
	AC/AC	24	20						
rs5812091 (dupC)	G/G	125	134	1.131 (0.658-1.942)	0.656	0.609 (0.106-3.496)	0.578	1.147 (0.708-1.859)	0.577
	G/GC	45	39						
	GC/GC	4	2						

HbA1c and TC were adjusted in three models. CI=confidence interval

Figure S1:

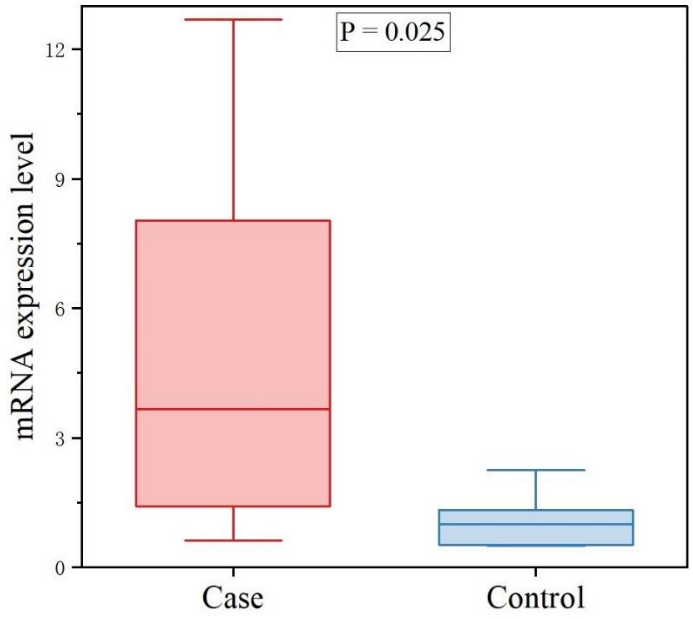

Figure S1. Boxplots showing THBS1 mRNA expression of the DR and DM patients. Center lines show the medians; box limits indicate the 25th and 75th percentiles; whiskers extend to the 5th and 95th percentiles.
